# The Interplay between Aquaporin-1 and the Hypoxia-Inducible Factor 1α in a Lipopolysaccharide-Induced Lung Injury Model in Human Pulmonary Microvascular Endothelial Cells

**DOI:** 10.3390/ijms231810588

**Published:** 2022-09-13

**Authors:** Chrysi Keskinidou, Nikolaos S. Lotsios, Alice G. Vassiliou, Ioanna Dimopoulou, Anastasia Kotanidou, Stylianos E. Orfanos

**Affiliations:** First Department of Critical Care Medicine & Pulmonary Services, Medical School, National and Kapodistrian University of Athens, Evangelismos Hospital, 10676 Athens, Greece

**Keywords:** AQP1, HIF1A, HPMEC, lung injury, LPS

## Abstract

Aquaporin-1 (AQP1), a water channel, and the hypoxia-inducible factor 1α (HIF1A) are implicated in acute lung injury responses, modulating among others pulmonary vascular leakage. We hypothesized that the AQP1 and HIF1A systems interact, affecting mRNA, protein levels and function of AQP1 in human pulmonary microvascular endothelial cells (HPMECs) exposed to lipopolysaccharide (LPS). Moreover, the role of AQP1 in apoptosis and wound healing progression was examined. Both AQP1 mRNA and protein expression levels were higher in HPMECs exposed to LPS compared to untreated HPMECs. However, in the LPS-exposed *HIF1A*-silenced cells, the mRNA and protein expression levels of AQP1 remained unaltered. In the permeability experiments, a statistically significant volume increase was observed at the 360 s time-point in the LPS-exposed HPMECs, while LPS-exposed *HIF1A*-silenced HPMECs did not exhibit cell swelling, implying a dysfunctional AQP1. AQP1 did not seem to affect cell apoptosis yet could interfere with endothelial migration and/or proliferation. Based on our results, it seems that *HIF1A* silencing negatively affects AQP1 mRNA and protein expression, as well as AQP1 function, in the setting of lung injury.

## 1. Introduction

Diffuse endothelial injury, intense promotion of the coagulation system, increased capillary permeability, hyaline membrane formation, and hypoxemia comprise the main pathological characteristics of acute respiratory distress syndrome (ARDS) [1,2]. ARDS is caused by a number of both direct and indirect lung injury etiologies, and most commonly develops in the setting of sepsis. Sepsis is characterized by a dysregulated immune response to the invading pathogens; when this response becomes overwhelming, sepsis-induced organ dysfunction can be established [3]. The administration of endotoxins is one of the most commonly used techniques for mimicking clinical sepsis in experimental models. Lipopolysaccharide (LPS), a glycolipid present in gram-negative bacteria’s outer membrane, is the most widely utilized endotoxin [4].

When injury is present in the lungs, the damage caused to the endothelium results in a diffuse inflammatory response and edema formation in the alveolar space. To eliminate the expansion of inflammation, endothelial cells become active, and alter their shape and function to facilitate the innate and adaptive immunity responses [5]. Endothelial leakage of protein-rich fluid from the capillary to the alveolar area causes hyper-inflammatory responses and disrupts the physiological osmotically-driven water transport in the lungs [6].

Aquaporin 1 (AQP1), a water channel, is expressed in the majority of endothelial cells and epithelial barriers in healthy humans [7]. In the respiratory system, AQP1 is expressed in the lung endothelium, in particular in the microvascular endothelium, and in pneumocytes [8]. AQP1 expression is modified in septic patients and patients with pulmonary diseases [9,10,11]. Moreover, AQP1 is also known to participate in a plethora of biological functions, namely cell proliferation, migration, apoptosis, and in inflammatory processes [12,13,14,15].

On the other hand, the degree of hypoxemia is the main parameter for the ARDS severity classification [2]. Hypoxia-inducible factors (HIFs) are transcription factors that, under hypoxic conditions, act as main mediators in the hypoxia response. In particular, the hypoxia-inducible factor 1α (HIF1A), is involved in acute lung injury responses, modulating pulmonary vascular leakage [16]. Bacteria and bacterial components promote the stabilization of the HIF system, resulting in the activation of HIF-dependent signaling pathways, including pro-inflammatory and immune responses [17]. HIF signaling and particularly HIF1A have been implicated in different phases of inflammatory responses during sepsis progression [18].

In this study, we aimed to investigate the interaction between AQP1 and HIF1A in lipopolysaccharide (LPS)-exposed human pulmonary microvascular endothelial cells (HPMECs). We examined how the silencing of *HIF1A* affects AQP1 mRNA and protein expression levels, as well as the water permeability function of AQP1. Additionally, we investigated whether, in our model, AQP1 is implicated in other biological processes, including cell apoptosis and wound healing, apart from its principal role as a water channel.

## 2. Results

### 2.1. HIF1A Silencing Abrogates the LPS-Induced Increase in AQP1 mRNA and Protein Expression

**Figure 1:**
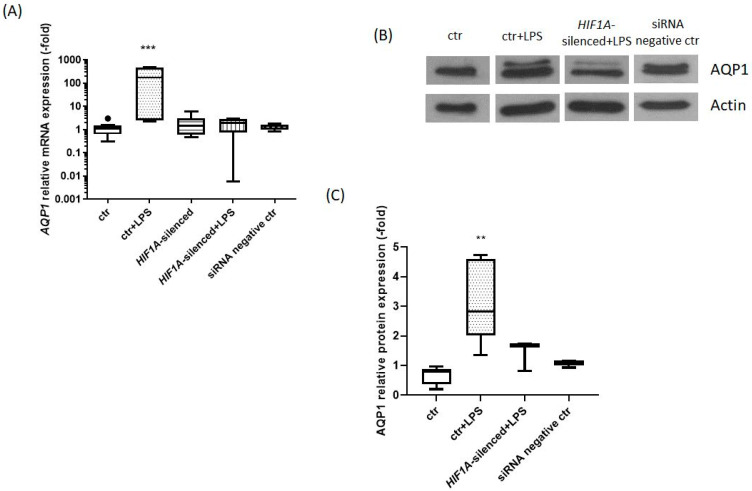
(**A**) *AQP1* relative mRNA expression, (**B**) Representative expression of AQP1 and actin proteins in LPS-exposed HPMECs and LPS-exposed *HIF1A*-silenced HPMECs, (**C**) AQP1 relative protein expression. (**A**) *AQP1* mRNA expression was measured in untreated HPMECs (ctr), *HIF1A*-silenced HPMECs, LPS-exposed HPMECs (ctr + LPS), *HIF1A*-silenced HPMECs exposed to LPS, and in HPMECs transfected with a universal scrambled negative control siRNA duplex (siRNA negative ctr). Data are presented as box plots. Analysis was performed by one-way ANOVA followed by the Kruskal Wallis posthoc test. Line in the middle, median value; lower and upper lines, 25th and 75th centiles; whiskers, range of values; ***, *p* < 0.001 compared to ctr. Results from 4 independent experiments. (**B**) Protein expression was analyzed by SDS-PAGE and immunoblotting. Relative protein expression was performed using densitometry with actin as a loading control. (**C**) AQP1 protein expression was measured in untreated HPMECs (ctr), LPS-exposed HPMECs (ctr + LPS), *HIF1A*-silenced HPMECs exposed to LPS, and in HPMECs transfected with a universal scrambled negative control siRNA duplex (siRNA negative ctr). Data are presented as box plots. Analysis was performed by one-way ANOVA followed by the Kruskal Wallis posthoc test. Line in the middle, median value; lower and upper lines, 25th and 75th centiles; whiskers, range of values; **, *p* < 0.01 compared to ctr. Results from 3 independent experiments.

*AQP1* mRNA expression levels were measured in untreated HPMECs (ctr), HPMECs exposed to LPS for 24 h, and *HIF1A*-silenced HPMECs either exposed or not to LPS. The changes in *AQP1* mRNA expression levels are presented in Figure 1A. *AQP1* mRNA levels in HPMECs exposed to LPS for 24 h were elevated 175-fold compared to untreated HPMECs (*p* < 0.001). However, in the *HIF1A*-silenced HPMECs, exposure to LPS did not affect *AQP1* mRNA expression (*p* > 0.05).

We also examined the protein expression levels of AQP1. The AQP1 relative protein expression is presented in Figure 1B,C. In HPMECs exposed to LPS for 24 h, the protein expression of AQP1 was statistically significantly elevated when compared to untreated HPMECs [2.83 (2.02–4.61) vs. 0.80 (0.79–0.96), respectively, *p* < 0.01). The AQP1 protein levels in the *HIF1A*-silenced HPMECs exposed to LPS were not significantly increased compared to untreated HPMECs [1.68 (0.83–1.74) vs. 0.80 (0.79–0.96), respectively, *p* > 0.05; Figure 1C].

### 2.2. Osmotic Challenge of Untreated and HIF1A-Silenced HPMECs Exposed to LPS

**Figure 2:**
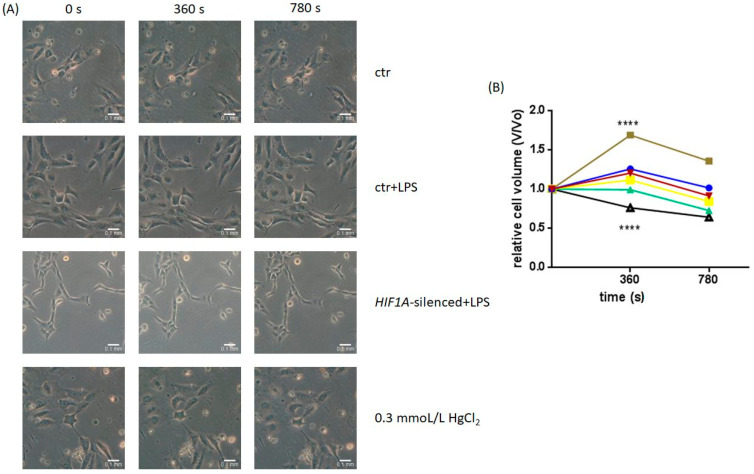
(**A**,**B**) Time-course of the osmotic swelling of HPMECs. (**A**) Representative images of the osmotically challenged untreated HPMECs (ctr), the LPS-exposed HPMECs (ctr + LPS), *HIF1A*-silenced HPMECs exposed to LPS, and HPMECs exposed to HgCl_2_, an AQP1 blocker (**B**) Time-course representation of the osmotically challenged untreated HPMECs (blue line with circles), *HIF1A*-silenced HPMECs (yellow line with squares), the LPS-exposed HPMECs (brown line with squares), *HIF1A*-silenced HPMECs exposed to LPS (green line with triangles), HPMECs exposed to 0.3 mmoL/L HgCl_2_ for 5 min (black line with triangles), HPMECs transfected with a universal scrambled negative control siRNA duplex (red line with triangles). Analysis was performed by 2-way ANOVA followed by Tukey’s posthoc test. ****, *p* < 0.0001 compared to ctr. Results from 4 independent experiments.

AQP1, as mentioned above, is a water channel and among others, its main function is water movement. Therefore, we examined whether LPS exposure and *HIF1A* silencing promote functional changes in HPMECs by interfering with AQP1 activity. The results of the permeability assay are presented in Figure 2. The HPMECs exposed to LPS exhibited a statistically significant cell volume increase compared to untreated HPMECs at the 360 s time-point (1.69 ± 0.11 vs. 1.26 ± 0.05, *p* < 0.0001), indicating an increase in AQP1 function. On the contrary in the LPS-exposed *HIF1A*-silenced HPMECs there was no increase in cell volume at the 360 s time-point (0.99 ± 0.09 vs. 1.26 ± 0.05, *p* > 0.05). When HPMECs were exposed to HgCl_2_ (0.3 mmoL/L for 5 min), an AQP1 blocker, the cells exhibited cell volume shrinkage compared to untreated HPMECs (0.76 ± 0.04 vs. 1.26 ± 0.05, *p* < 0.0001), as expected.

### 2.3. The Role of AQP1 in Apoptosis

**Figure 3:**
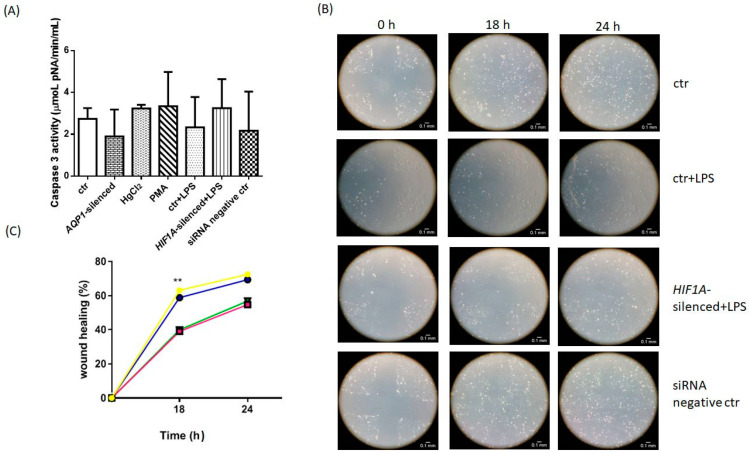
(**A**) Caspase 3 activity, (**B**,**C**) Time-course of wound healing of HPMECs. (**A**) In order to examine apoptosis in HPMECs, caspase 3 activity was measured in untreated HPMECs (ctr), *AQP1*-silenced HPMECs, HPMECs exposed to 0.3 mmoL/L HgCl_2_ for 5 min, HPMECs exposed to 100 nM PMA for 2 h, LPS-exposed HPMECs (ctr + LPS), LPS-exposed *HIF1A*-silenced HPMECs and HPMECs transfected with a universal scrambled negative control siRNA duplex. Data are presented as boxes. Each box represents the mean values and the bars represent the standard deviation values. Analysis was performed by one-way ANOVA followed by Kruskal Wallis. Results from 3 independent experiments. (**B**,**C**) Time-course representation of the percentage of wound closure measured in untreated HPMECs (yellow line with circles), LPS-exposed HPMECs (pink line with squares), *HIF1A*-silenced HPMECs exposed to LPS (green line with triangles), and HPMECs transfected with a universal scrambled negative control siRNA duplex (blue line with circles). **, *p* < 0.01 compared to untreated cells. Analysis was performed by 2-way ANOVA followed by the Wilcoxon test. Results from 3 independent experiments.

We next aimed to examine whether dysregulated AQP1 expression affects apoptosis in HPMECs. Therefore, we measured the activity of caspase 3 in untreated HPMECs, HPMECs exposed to LPS, *AQP1*-silenced HPMECs, HPMECs exposed to either the AQP1 inducer, phorbol 12-myristate 13-acetate (PMA), or its inhibitor HgCl_2_, and in *HIF1A*-silenced HPMECs exposed to LPS. As presented in Figure 3A, we observed no statistically significant caspase 3 activity difference between the conditions examined. The results are presented in Table 1. From the results, we can conclude that dysregulated AQP1 expression does not have any impact on apoptosis in our human pulmonary microvascular endothelium cell line.

### 2.4. Wound Healing Assay

The impact of AQP1 was explored in a wound healing assay. Alongside, we also examined whether LPS exposure and *HIF1A* silencing interfere with the postulated role of AQP1 in wound healing. The migration rate was calculated as the percentage of wound closure over time. Eighteen (18) hours following wound creation, the untreated HPMECs exhibited 63% wound closure, while at 24 h closure reached 72%. The rate of wound closure in the HPMECs treated with LPS and the LPS-exposed *HIF1A*-silenced HPMECs, at 18 h was 39% and 40%, respectively, while at 24 h closure reached 55% and 57%, respectively. Hence, as seen in Table 2 and Figure 3B,C, LPS-exposed and *HIF1A*-silenced HPMECs exhibited a slower wound healing rate compared to untreated HPMECs (*p* < 0.01).

## 3. Discussion

In the present study, we demonstrated that the AQP1 and HIF1A systems interact under inflammatory conditions. The exposure of HPMECs to LPS resulted in an upregulation of the mRNA and protein expression of AQP1. Moreover, LPS treatment increased the relative cell volume when compared to untreated HPMECs. However, in the LPS-exposed *HIF1A*-silenced HPMECs, no increase in the mRNA and protein expression of AQP1 was observed. In the same manner, at the functional level, silencing of *HIF1A* once again abolished the effect of LPS on the osmotic cell challenge. Our results could indicate a possible role of *HIF1A* in the LPS-induced changes observed in the mRNA and protein expression levels and function of AQP1.

LPS is a component of the outer membrane of Gram-negative bacteria and a known immunostimulatory agent inducing pro-inflammatory responses [19]. Many experimental studies have examined how LPS affects AQP1 expression. Based on the results of these studies, it can be concluded that AQP1 is differentially regulated by LPS [8,20,21,22,23]. In our study, the 24 h exposure of HPMECs to LPS resulted in induction of both mRNA and protein expression, and function of AQP1, suggesting that LPS may act as an upregulator of AQP1 under inflammatory stimuli in pulmonary microvascular endothelial cells. 

Inflammatory stimuli such as bacteria and bacterial components, including LPS, are known to upregulate HIF1A. HIFs are transcription factors that under normoxia are inactivated; however, under hypoxic conditions become activated. In particular, hypoxia enables HIF1A stabilization, enabling it to form heterodimers with the HIF1B subunit. In the nucleus, the heterodimeric transcription factor HIF1A/HIF1B binds to promoters containing the hypoxia-response element (HRE), inducing the HIF-dependent transcription of target genes [24,25].

Several studies, examining both in vitro and in vivo septic lung inflammation models, revealed that following lung injury HIF1A is activated in endothelial cells; hence, inducing HIF signaling pathways has a protective role, by promoting cell proliferation, vascular repair, and resolution of inflammation. These studies imply a potential treatment strategy based on the therapeutic activation of HIF1A signaling pathways [26,27,28]. Other studies using in vitro and in vivo models of LPS-induced lung injury, have suggested that inhibiting HIF1A activity and the related signaling pathways could provide therapeutic effects on acute lung injury [29,30,31,32]. Our results showed that silencing of *HIF1A* in HPMECs seems to abrogate the LPS-induced AQP1 upregulation. *HIF1A*-silenced HPMECs exposed to LPS did not exhibit increased mRNA and protein expression of AQP1, indicating a possible interplay between the two systems.

The role of AQP1 in water permeability and edema formation, and the effect of *HIF1A* silencing on the hypoxia-induced AQP1 upregulation was studied in an in vitro model of primary cells [33]. To examine the role of AQP1 in water transport, Zhang et al. either silenced or overexpressed *AQP1*. Silencing of *AQP1* caused cell shrinkage, whereas *AQP1* overexpression caused cell swelling [33]. These results agree with our findings; exposure of cells to LPS promoted cell swelling, while treatment with HgCl_2_, an AQP1 blocker, promoted cell shrinkage. Moreover, Zhang et al. investigated the involvement of HIF1A in AQP1 regulation. In agreement with our results, they concluded that silencing of *HIF1A* decreased the hypoxia-induced mRNA and protein AQP1 upregulation. In our study, we also demonstrated that *HIF1A* silencing abrogated the LPS-induced increase in AQP1 mRNA and protein levels, and the water permeability function of AQP1. 

Many studies have underlined the importance of AQP1 in cell survival and cell migration. However, the exact role of AQP1 in the abovementioned processes differs based on the model examined. In an in vitro model of septic acute kidney injury, overexpression of AQP1 promoted cell viability and reduced apoptosis [34], while in Chinese hamster ovary K1 cells, AQP1 overexpression induced apoptosis [35]. In human pulmonary artery smooth muscle cells, silencing of AQP1 decreased proliferation and migration ability and induced apoptosis, while the overexpression of AQP1 enhanced the migration and proliferation rates [36]. In our model, based on our results that AQP1 is upregulated by LPS treatment, we examined whether AQP1 upregulation or inhibition affects apoptosis in HPMECs. Caspase 3 activity remained unaltered in both HPMECs exposed to LPS and/or PMA, and in HPMECs silenced for *AQP1* or exposed to HgCl_2_*,* compared to untreated HPMECs. Moreover, the apoptosis rate remained stable in the HPMECs silenced for *HIF1A* and treated with LPS. We conclude that in our model, AQP1 does not affect cell apoptosis.

Endothelial cell migration is important in injury repair and vascular integrity maintenance, while it also occurs during vasculogenesis and angiogenesis [37]. AQP1 expression has been linked to angiogenesis and tumor migration in different types of cancer, including lung cancer, in in vivo and in vitro experiments [38,39,40,41,42,43]. Moreover, the effect of LPS on HPMEC migration is dose-dependent [44]. In our model, in the LPS-exposed HPMECs and the LPS-exposed *HIF1A*-silenced HPMECs, wound closure was slower than the closure rate observed in untreated HPMECs. This might suggest that in pulmonary microvascular endothelial cells, overexpression of AQP1 possibly interferes with the processes of endothelial migration and/or proliferation, which could result in the loss of vascular integrity.

## 4. Materials and Methods

Since the pulmonary endothelium has been recognized as a key modulator of lung disorders, such as ARDS, we performed our experiments on human pulmonary microvascular endothelial cells. It should be noted that widespread vascular endothelial injury is thought to be a major mechanism for multiorgan dysfunction in sepsis, and pulmonary microvascular endothelial injury in ARDS [4]. The cell line used for the experiments was the HPMEC-ST1.6R, which was a generous gift from Dr. Ronald E. Unger, Johannes Gutenberg-Universität Mainz [45]. The SiTran2.0 transfection reagent from Origene (Origene, Rockville, MD, USA) was used. The transfection assay was performed according to the manufacturer’s instructions. Prior to LPS (Sigma-Aldrich, St Louis, MO, USA) exposure, cells were starved for 2 h in serum-free culture media. Freshly prepared LPS was diluted to a final concentration of 100 ng/mL in serum-free culture media. Cells were incubated for 24 h in the presence of LPS.

RNA was extracted, using the TRI reagent (Sigma-Aldrich, Saint Louis, MO, USA), reverse transcribed (Nippon Genetics, Düren, Germany), and the resulting cDNA was used as a template for the real-time PCR analysis (Kapa SYBR^®^ Fast PCR Master Mix, Merck KGaA, Darmstadt, Germany), which was carried out on a BioRad CFX Connect thermocycler (Bio-Rad Laboratories, Inc., Hercules, CA, USA). Primers were designed for specific genes using information from the NCBI Sequence database. The primers’ sequences are listed in Table 3. The untreated cells were used as a calibrator, and the relative quantification of the expression analysis of treated cells was performed using the comparative CT method 2^−^^ΔΔCT^ [46]. The expression of the housekeeping gene, *GAPDH* was used to normalize the levels of expression of the target genes.

Using a hand-held homogenizer, cells were homogenized on ice in phosphate-buffered saline (PBS) with the addition of 1 mM dithiothreitol (DTT) (Sigma-Aldrich, St Louis, MO, USA), 1 mM phenylmethylsulfonyl fluoride (PMSF) (Sigma-Aldrich, St Louis, MO, USA) in isopropanol, and 1% protease inhibitors (Sigma-Aldrich, St Louis, MO, USA). The homogenate was centrifuged at 4 °C for 5 min at 1000× *g*, and the resultant supernatant, titled “total protein,” was collected and stored at −80 °C until used. For the total protein concentration determination, the bicinchoninic acid (BCA) method [47] was used. SDS-polyacrylamide gel electrophoresis was performed on 15% polyacrylamide slab gels on a “Biorad Mini Protean II” electrophoresis apparatus (Bio-Rad Laboratories, Inc., Hercules, CA, USA), followed by western transfer onto an Immobilon-P PVDF membrane (0.45 μL pore size, MilliporeSigma, Burlington, MA, USA). Immunological detection of AQP1 was performed with a polyclonal antibody from MilliporeSigma (MilliporeSigma, Burlington, MA, USA). Protein detection was performed using enhanced chemiluminescence (ECL) (MilliporeSigma, Burlington, MA, USA), while the ImageJ 1.52a software (National Institutes of Health, Madison, WI, USA) [48] was used to determine relative protein expression using densitometry with actin as a loading control.

Osmotic swelling was measured as previously reported [11]. HPMECs were transferred in a low osmolarity solution and photos were collected at 1-min intervals for a total of 780 s, or until HPMEC rupture occurred. The relative volume V/Vo was calculated as the ratio of measured volume at various time points during the osmotic challenge to the baseline volume. The ImageJ software was used to calculate volume changes.

Apoptosis was measured using the caspase 3 kit from Sigma-Aldrich (Sigma-Aldrich, Saint Louis, MO, USA), following the manufacturer’s instructions. Briefly, cells were incubated in the presence of 1 μg/mL staurosporine (Cayman Chemical, Ann Arbor, MI, USA) for 3 h to induce apoptosis, and cell lysates were collected for the measurement of caspase 3 activity.

Twenty-eight thousand (28,000) HPMECs were seeded in 24-well cell culture plates (60–70% confluency the next day). When cells reached 100% confluence, a wound in the shape of a cross was created using a 200 μL pipette tip. Cells were then washed once with PBS to remove cell debris and were observed on a Zeiss Axiovert 25-phase contrast microscope (Carl Zeiss AG, Oberkochen, Germany) when the first image was retrieved (time-point 0 h) using a Canon digital camera (Canon Inc., Ota City, Tokyo, Japan). The second image was retrieved after 18 h, while the last image was retrieved after 24 h. Images were analyzed using the ImageJ software. The migration rate was expressed as the percentage of wound closure over time. 

Data are presented either as means ± standard deviation (SD) or medians with the interquartile range (IQR), accordingly. Statistical analysis was performed by one-way or 2-way ANOVA followed by Kruskal Wallis, Tukey’s, or Wilcoxon tests, as appropriate. For the statistical analysis, the GraphPad Prism 6 for Windows (GraphPad Software, San Diego, CA, USA) statistical program was used. *p*-values under 0.05 were considered significant.

## 5. Conclusions

Our results indicate a possible role of *HIF1A* in the LPS-induced changes observed in the mRNA and protein expression levels and function of AQP1, in the setting of lung injury. A better understanding of the emerging roles of AQP1 in both physiological and pathological lung processes could mark the beginning of the investigation of new therapeutic strategies, which could eliminate the overwhelming edema formation and inflammatory responses induced by the infectious injury present in the lungs.

## Figures and Tables

**Table 1:** Caspase 3 activity in HPMECs.

Sample	Caspase 3 Activity (μmoL pNA/min/mL)
ctr	2.74 ± 1.4
*AQP1*-silenced	1.90 ± 1.3
HgCl_2_	3.24 ± 0.18
PMA	3.34 ± 1.6
ctr + LPS	2.33 ± 1.5
*HIF1A*-silenced + LPS	2.77 ± 1.6
siRNA negative ctr	2.18 ± 1.9

Mean values with the standard deviation are presented. Analysis was performed by one-way ANOVA followed by Kruskal Wallis. Results from 3 independent experiments. ctr = untreated HPMECs.

**Table 2:** Wound healing assay in HPMECs.

Sample	18 h (%)	24 h (%)
ctr	63 ± 20	72 ± 19
ctr + LPS	39 ± 17 **	55 ± 20
*HIF1A*-silenced + LPS	40 ± 16 **	57 ± 19
siRNA negative ctr	59 ± 22	69 ± 20

Mean values with the standard deviation are presented. **, *p* < 0.01 compared to untreated HPMECs (ctr). Analysis was performed by 2-way ANOVA followed by the Wilcoxon test. Results from 3 independent experiments.

**Table 3:** Sequences of the primers used and the amplicon sizes.

Gene	Forward Primer	Reverse Primer	Amplicon Size (Base Pairs)
*AQP1*	5′-CTG-GGC-ATC-GAG-ATC-ATC-GG-3′	5′-ATC-CCA-CAG-CCA-GTG-TAG-TCA-3′	158
*HIF1A*	5′-GGC-GCG-AAC-GAC-AAG-AAA-AAG-3′	5′-CCT-TAT-CAA-GAT-GCG-AAC-TCA-CA-3′	154
*GAPDH*	5′-ATG-GGG-AAG-GTG-AAG-GTC-G-3′	5′-TAC-ATG-AGG-GCA-CGG-AAG-ATG-3′	108

## Data Availability

Data available upon reasonable request.

## References

[1] Bernard G.R., Artigas A., Brigham K.L., Carlet J., Falke K., Hudson L., Lamy M., Legall J.R., Morris A., Spragg R. (1994). The American-European Consensus Conference on ARDS. Definitions, mechanisms, relevant outcomes, and clinical trial coordination. Am. J. Respir. Crit. Care Med..

[2] Force T.A.D.T. (2012). Acute Respiratory Distress Syndrome: The Berlin Definition. JAMA.

[3] Singer M., Deutschman C.S., Seymour C.W., Shankar-Hari M., Annane D., Bauer M., Bellomo R., Bernard G.R., Chiche J.D., Coopersmith C.M. (2016). The Third International Consensus Definitions for Sepsis and Septic Shock (Sepsis-3). JAMA.

[4] Keskinidou C., Vassiliou A.G., Dimopoulou I., Kotanidou A., Orfanos S.E. (2022). Mechanistic Understanding of Lung Inflammation: Recent Advances and Emerging Techniques. J. Inflamm. Res..

[5] Vassiliou A.G., Kotanidou A., Dimopoulou I., Orfanos S.E. (2020). Endothelial Damage in Acute Respiratory Distress Syndrome. Int. J. Mol. Sci..

[6] Chalmers S., Khawaja A., Wieruszewski P.M., Gajic O., Odeyemi Y. (2019). Diagnosis and treatment of acute pulmonary inflammation in critically ill patients: The role of inflammatory biomarkers. World J. Crit. Care Med..

[7] Mobasheri A., Marples D. (2004). Expression of the AQP-1 water channel in normal human tissues: A semiquantitative study using tissue microarray technology. Am. J. Physiol. Cell Physiol..

[8] Vassiliou A.G., Manitsopoulos N., Kardara M., Maniatis N.A., Orfanos S.E., Kotanidou A. (2017). Differential Expression of Aquaporins in Experimental Models of Acute Lung Injury. In Vivo.

[9] Calero C., López-Campos J.L., Izquierdo L.G., Sánchez-Silva R., López-Villalobos J.L., Sáenz-Coronilla F.J., Arellano-Orden E., Montes-Worboys A., Echevarría M. (2014). Expression of aquaporins in bronchial tissue and lung parenchyma of patients with chronic obstructive pulmonary disease. Multidiscip. Respir. Med..

[10] Galán-Cobo A., Arellano-Orden E., Sánchez Silva R., López-Campos J.L., Gutiérrez Rivera C., Gómez Izquierdo L., Suárez-Luna N., Molina-Molina M., Rodríguez Portal J.A., Echevarría M. (2018). The Expression of AQP1 IS Modified in Lung of Patients with Idiopathic Pulmonary Fibrosis: Addressing a Possible New Target. Front. Mol. Biosci..

[11] Vassiliou A.G., Maniatis N.A., Orfanos S.E., Mastora Z., Jahaj E., Paparountas T., Armaganidis A., Roussos C., Aidinis V., Kotanidou A. (2013). Induced expression and functional effects of aquaporin-1 in human leukocytes in sepsis. Crit. Care.

[12] Verkman A.S., Matthay M.A., Song Y. (2000). Aquaporin water channels and lung physiology. Am. J. Physiol. Lung Cell. Mol. Physiol..

[13] Li C., Wang W. (2017). Molecular Biology of Aquaporins. Adv. Exp. Med. Biol..

[14] Verkman A.S. (2006). Aquaporins in endothelia. Kidney Int..

[15] Meli R., Pirozzi C., Pelagalli A. (2018). New Perspectives on the Potential Role of Aquaporins (AQPs) in the Physiology of Inflammation. Front. Physiol..

[16] Shimoda L.A., Semenza G.L. (2011). HIF and the lung: Role of hypoxia-inducible factors in pulmonary development and disease. Am. J. Respir. Crit. Care Med..

[17] Nizet V., Johnson R.S. (2009). Interdependence of hypoxic and innate immune responses. Nat. Rev. Immunol..

[18] Vanderhaeghen T., Vandewalle J., Libert C. (2020). Hypoxia-inducible factors in metabolic reprogramming during sepsis. FEBS J..

[19] Lu Y.C., Yeh W.C., Ohashi P.S. (2008). LPS/TLR4 signal transduction pathway. Cytokine.

[20] Hasan B., Li F.S., Siyit A., Tuyghun E., Luo J.H., Upur H., Ablimit A. (2014). Expression of aquaporins in the lungs of mice with acute injury caused by LPS treatment. Respir. Physiol. Neurobiol..

[21] Su X., Song Y., Jiang J., Bai C. (2004). The role of aquaporin-1 (AQP1) expression in a murine model of lipopolysaccharide-induced acute lung injury. Respir. Physiol. Neurobiol..

[22] Liu L.D., Wu X.Y., Tao B.D., Wang N., Zhang J. (2016). Protective effect and mechanism of hydrogen treatment on lung epithelial barrier dysfunction in rats with sepsis. Genet. Mol. Res. GMR.

[23] Wang X., Zhou X., Xia X., Zhang Y. (2021). Estradiol attenuates LPS-induced acute lung injury via induction of aquaporins AQP1 and AQP5. Eur. J. Inflamm..

[24] Wenger R.H., Stiehl D.P., Camenisch G. (2005). Integration of oxygen signaling at the consensus HRE. Sci. STKE Signal Transduct. Knowl. Environ..

[25] Evans C.E. (2022). Hypoxia-Inducible Factor Signaling in Inflammatory Lung Injury and Repair. Cells.

[26] McClendon J., Jansing N.L., Redente E.F., Gandjeva A., Ito Y., Colgan S.P., Ahmad A., Riches D.W.H., Chapman H.A., Mason R.J. (2017). Hypoxia-Inducible Factor 1α Signaling Promotes Repair of the Alveolar Epithelium after Acute Lung Injury. Am. J. Pathol..

[27] Huang X., Zhang X., Zhao D.X., Yin J., Hu G., Evans C.E., Zhao Y.Y. (2019). Endothelial Hypoxia-Inducible Factor-1α Is Required for Vascular Repair and Resolution of Inflammatory Lung Injury through Forkhead Box Protein M1. Am. J. Pathol..

[28] Wu G., Xu G., Chen D.-W., Gao W.-X., Xiong J.-Q., Shen H.-Y., Gao Y.-Q. (2018). Hypoxia Exacerbates Inflammatory Acute Lung Injury via the Toll-Like Receptor 4 Signaling Pathway. Front. Immunol..

[29] Peyssonnaux C., Cejudo-Martin P., Doedens A., Zinkernagel A.S., Johnson R.S., Nizet V. (2007). Cutting edge: Essential role of hypoxia inducible factor-1alpha in development of lipopolysaccharide-induced sepsis. J. Immunol..

[30] Yeh C.H., Cho W., So E.C., Chu C.C., Lin M.C., Wang J.J., Hsing C.H. (2011). Propofol inhibits lipopolysaccharide-induced lung epithelial cell injury by reducing hypoxia-inducible factor-1alpha expression. Br. J. Anaesth..

[31] Xu M., Cao F., Liu L., Zhang B., Wang Y., Dong H., Cui Y., Dong M., Xu D., Liu Y. (2011). Tanshinone IIA-induced attenuation of lung injury in endotoxemic mice is associated with reduction of hypoxia-inducible factor 1α expression. Am. J. Respir. Cell Mol. Biol..

[32] Li X., Shan C., Wu Z., Yu H., Yang A., Tan B. (2020). Correction to: Emodin alleviated pulmonary inflammation in rats with LPS-induced acute lung injury through inhibiting the mTOR/HIF-1α/VEGF signaling pathway. Inflamm. Res..

[33] Zhang J., Xiong Y., Lu L.X., Wang H., Zhang Y.F., Fang F., Song Y.L., Jiang H. (2013). AQP1 expression alterations affect morphology and water transport in Schwann cells and hypoxia-induced up-regulation of AQP1 occurs in a HIF-1alpha-dependent manner. Neuroscience.

[34] Wang Y., Zhang W., Yu G., Liu Q., Jin Y. (2018). Cytoprotective effect of aquaporin 1 against lipopolysaccharide-induced apoptosis and inflammation of renal epithelial HK-2 cells. Exp. Ther. Med..

[35] Jablonski E.M., Webb A.N., McConnell N.A., Riley M.C., Hughes F.M. (2004). Plasma membrane aquaporin activity can affect the rate of apoptosis but is inhibited after apoptotic volume decrease. Am. J. Physiol. Cell Physiol..

[36] Schuoler C., Haider T.J., Leuenberger C., Vogel J., Ostergaard L., Kwapiszewska G., Kohler M., Gassmann M., Huber L.C., Brock M. (2017). Aquaporin 1 controls the functional phenotype of pulmonary smooth muscle cells in hypoxia-induced pulmonary hypertension. Basic Res. Cardiol..

[37] Michaelis U.R. (2014). Mechanisms of endothelial cell migration. Cell. Mol. Life Sci..

[38] Saadoun S., Papadopoulos M.C., Hara-Chikuma M., Verkman A.S. (2005). Impairment of angiogenesis and cell migration by targeted aquaporin-1 gene disruption. Nature.

[39] Dorward H.S., Du A., Bruhn M.A., Wrin J., Pei J.V., Evdokiou A., Price T.J., Yool A.J., Hardingham J.E. (2016). Pharmacological blockade of aquaporin-1 water channel by AqB013 restricts migration and invasiveness of colon cancer cells and prevents endothelial tube formation in vitro. J. Exp. Clin. Cancer Res..

[40] Papadopoulos M.C., Saadoun S., Verkman A.S. (2008). Aquaporins and cell migration. Pflug. Arch. Eur. J. Physiol..

[41] Jiang Y. (2009). Aquaporin-1 activity of plasma membrane affects HT20 colon cancer cell migration. IUBMB Life.

[42] Wei X., Dong J. (2015). Aquaporin 1 promotes the proliferation and migration of lung cancer cell in vitro. Oncol. Rep..

[43] Wagner K., Unger L., Salman M.M., Kitchen P., Bill R.M., Yool A.J. (2022). Signaling Mechanisms and Pharmacological Modulators Governing Diverse Aquaporin Functions in Human Health and Disease. Int. J. Mol. Sci..

[44] Zheng X., Zhang W., Hu X. (2018). Different concentrations of lipopolysaccharide regulate barrier function through the PI3K/Akt signalling pathway in human pulmonary microvascular endothelial cells. Sci. Rep..

[45] Krump-Konvalinkova V., Bittinger F., Unger R.E., Peters K., Lehr H.-A., Kirkpatrick C.J. (2001). Generation of Human Pulmonary Microvascular Endothelial Cell Lines. Lab. Investig..

[46] Livak K.J., Schmittgen T.D. (2001). Analysis of relative gene expression data using real-time quantitative PCR and the 2(-Delta Delta C(T)) Method. Methods.

[47] Lleu P.L., Rebel G. (1991). Interference of Good’s buffers and other biological buffers with protein determination. Anal. Biochem..

[48] Schneider C.A., Rasband W.S., Eliceiri K.W. (2012). NIH Image to ImageJ: 25 years of image analysis. Nat. Methods.

